# Network Pharmacology-Based Analysis of *Pogostemon cablin* (Blanco) Benth Beneficial Effects to Alleviate Nonalcoholic Fatty Liver Disease in Mice

**DOI:** 10.3389/fphar.2021.789430

**Published:** 2021-11-24

**Authors:** Yizhe Cui, Qiuju Wang, Renxu Chang, Ahmad Aboragah, Juan J. Loor, Chuang Xu

**Affiliations:** ^1^ College of Animal Science and Technology, Heilongjiang Bayi Agricultural University, Daqing, China; ^2^ Heilongjiang Provincial Key Laboratory of Prevention and Control of Bovine Diseases, Heilongjiang Bayi Agricultural University, Daqing, China; ^3^ Department of Animal Sciences, Division of Nutritional Sciences, University of Illinois, Urbana, IL, United States

**Keywords:** network pharmacology, *Pogostemon cablin* (Blanco) Benth, mice, AML12 cells, multi-targets, NAFLD

## Abstract

Nonalcoholic fatty liver disease (NAFLD) is the most common cause of chronic liver disease and is associated with high morbidity and mortality. *Pogostemon cablin* (Blanco) Benth/Huo Xiang (HX) is a perennial herb with unique anti-oxidant and anti-inflammatory properties, and thus, can positively affect liver function. In this study, we used network pharmacology to predict the potential mechanism of HX on NAFLD. Pharmacological experiments were used to verify the effect of HX on the functions of NAFLD. Network pharmacology identified nine components that interacted with 82 NAFLD-related targets, revealing four target genes: *TNF*, *IL6*, *TP53*, and *AKT1*. HX prevents the development and progression of NAFLD through different pathways and targets with quercetin-regulated lipid metabolism, anti-inflammatory, and anti-oxidant pathways playing an essential role in the treatment of NAFLD. Compared with feeding HFD, HX significantly attenuated lipid accumulation *in vivo* with mice and also *in vitro* with mouse liver cells. A high dose of HX decreased hepatocyte lipid accumulation and the abundance of SREBF1 and FASN. Validation experiments revealed that HX inhibited the activation of NF-κB/IκB signaling and decreased the release and levels of pro-inflammatory factors (TNF-α and IL-6). These data suggest that HX can attenuate abnormal lipid metabolic responses and enhance antioxidant mechanisms. Thus, the pharmacological effects from plants used in traditional Chinese medicine are achievde through a multi-level response.

## Introduction

Nonalcoholic fatty liver disease (NAFLD) is a crucial component of the metabolic syndrome when obesity and insulin resistance (IR) are present (Danford and Lai, 2019). Inflammatory reactions induced by reactive oxygen species (ROS) in liver parenchymal cells during NAFLD characterize the so-called “first hit” (Buzzetti et al., 2016). Dysregulation of adipocyte metabolism in the metabolic syndrome is an independent risk factor for developing NAFLD (Eslam and George, 2019). Natural substances are not the only effective treatment for obesity, diabetes, insulin resistance (IR), and other metabolic diseases, but they are relatively safe to consume (Li et al., 2016). Traditional Chinese medicine (TCM) formulas based on plant extracts contain substances capable of eliciting the so-called “multiple organ-multiple hit” effect (Yan et al., 2020). Various TCMs and supplements offer suitable therapeutic options for the treatment and prevention of NAFLD (Perumpail et al., 2018).

Patchouli (*Pogostemon cablin* (Blanco) Benth./Huo Xiang (HX)), from the Labiatae family, has been used by humans for the treatment of anorexia, vomiting, hepatic injury (Cui BW. et al., 2019), and other intestinal disorders (Tuan et al., 2012; Li HQ. et al., 2013). Compilation of Materia Medica (“Ben Cao Gang Mu” in pinyin) also contains information indicating that HX can be used to treat dampness obstruction, abdominal distension, vomiting, and heat dampness syndrome. Previous studies indicated that HX exerts anticarcinogenic (Lu et al., 2016) and protective effects against lung (Su et al., 2016) and brain injury (Wei et al., 2018). Extracts of HX are also uesed to treat inflammatory (Li et al., 2011) and oxidative stress-induced disorders (Lian et al., 2018). In addition, HX reduces toll-like receptor (TLR) 4 and glycosylation end product (receptor for advanced glycation end products [RAGE]) protein signaling contributing to lipopolysaccharide-induced liver injury (Cho et al., 2015). Despite the substantial amount of information available, the main pharmacodynamic components contained in HX and the molecular mechanism of its protective effect on acute alcoholic liver injury are still unclear.

Network pharmacology is an emerging discipline based on systems biology theory, i.e. combining multiple disciplines to design multi-target drugs, analyze the interactions between biological networks, and identify desired targets (Hao and Xiao, 2014). It provides a scientific channel for mechanistic research focused on TCM prescriptions and how they may be successfully applied to various diseases (Luo et al., 2020). The framework of system biology is consistent with the characteristics of multi-component, multi-target, and multi-channel interactions of a given compound. The systems approach is compatible with the holistic view to differentiate various syndromes, but alos their treatment using TCM.

Therefore, our goal was to apply a network pharmacology–based approach to investigate the relationship between HX compounds and potential targets in NAFLD. Our specific objectives were to induce NAFLD *in vivo* and *in vitro* to study the underlying mechanisms whereby HX could positively affect the disease, focusing on the use of network pharmacology and experimental validation.

## Materials and Methods

### Network Pharmacology Study

#### Collection of Potential Targets of HX

A search was performed for the active components of HX in the TCM system pharmacology technology platform (TCMIP, http://www.tcmip.cn/TCMIP/index.php/Home/) databases with “ *Pogostemon cablin* (Blanco) Benth” as the keyword. The bioactive compounds were screened further using oral bioavailability (OB) criteria of ≥30% and drug-likeness (DL) of ≥0.18 (Xu et al., 2019). Potential bioactive compounds of HX were submitted to the DrugBank (https://www.drugbank.ca/) server. Then, potential targets (proteins) from DrugBank and the Traditional Chinese Medicine Systems Pharmacology (TCMSP) database were translated into genes using STRING 11.5 (https://string-db.org/). The UniProt (https://www.uniprot.org/) database was used to standardize the results. An interaction network of component targets was constructed and visualized via Cytoscape software.

Nonalcoholic fatty liver–related targets were mined from DisGeNET (https://www.disgenet.org/) (Piñero et al., 2021). We used “nonalcoholic fatty liver disease” as index words and limited the species to “Homo sapiens” to collect therapeutic targets for NAFLD. Lastly, Cytoscape 3.8.2 was used to perform visual network analysis of the “disease-target.”

#### Construction and Analysis of the Protein-Protein Interaction Network

To explain the interactions between putative targets, the target information between HX and NAFLD obtained above was imported into the STRING platform (https://string-db.org) to generate relationships between these potential targets. High-confidence data >0.4 were included to ensure reliability of the analysis. Subsequently, the protein–protein interaction network was constructed and visualized.

#### Functional Enrichment and Pathways Analysis

The potential targets for the active ingredients of HX in NAFLD were annotated using the biomolecular function of Metascape (https://metascape.org/gp/index.html#/main/step1). A heatmap was plotted using http://www.bioinformatics.com.cn, a free online platform for data analysis and visualization. Gene Ontology (GO) enrichment analyses included biological process (BP), cellular component (CC), and molecular function (MF). In addition, Kyoto Encyclopedia of Gene and Genome (KEGG) pathway enrichment analyses were performed. Lastly, *p* < 0.01 was set as the threshold value to identify biological processes and signaling pathways that were significantly different. Enrichment analyses of the KEGG for the ingredients of HX in NAFLD were analyzed using ClueGO.

### Herbal Plant Extract

The HX used in this study (Origin: Guangxi, China; Batch No.: 20170601; Quality standard: Chinese Pharmacopoeia 2015) was purchased from Fu Rui Bang Chinese Medicine Co., Ltd. (Daqing, China). Raw herbs were soaked in distilled water overnight, followed by decoction twice in boiling water (60 min each time). The combined aqueous extract was filtered through gauze and then heated until evaporation (Gou et al., 2017). Low-speed centrifugation was used to remove insoluble particles, and the concentration of the HX residue was at 1 g/ml. The supernatant was sterilized by filtration through a 0.22-μm Millipore filter (Millex, GP) and stored at 4°C until use. The main components of HX were analyzed by high-performance liquid chromatography-electrospray ionization/mass spectrometry (HPLC-ESI/MS Qingdao Kechuang Quality Inspection Co., Ltd. Qingdao, China) (Application No. PDFD-16-01 D/0). All samples were extracted with 300 µl methanol and 10 µl of internal standard was added. Samples were then sonicated in an ice bath. Two-hundred μl supernatant was transferred to vials for LC-MS analysis. The data were extracted and preprocessed using compound discovery software (Thermo), and then normalized and edited into a two-dimensional data matrix using Excel 2010 software, including retention time (RT), compound molecular weight (COMP MW), observed value (sample), and peak strength (Cui et al., 2019c).

### Animals and Treatment

Male ICR mice (20–22 g; 8 weeks) were obtained from Harbin Medical University (Daqing, China). The mice were housed in cages with a 12-h light/dark cycle in a temperature-controlled environment and were acclimatized to laboratory conditions for 1 week before the study. Subsequently, mice were randomly divided into five groups of six animals each: 1) a control (Con) group and 2) a NAFLD group fed a high-fat diet (HFD), 3) a low-dose group fed HFD +1.8 g/kg HX (HFD+1.8HX) given orally (0.1 ml per 10 g body weight), 4) a medium-dose group (HFD+4.5HX), and a high-dose group (HFD+9.0HX). The dosage of HX was chosen based on recommendations from the Chinese Pharmacopoeia (Chinese Pharmacopoeia Commission, 2015). According to the literature, HX does not cause any behavioral changes or mortality (Li CW. et al., 2013). The Con diet was prepared by following the AIN-93M formula with adjustments (Reeves et al., 1993, 93), and the HFD was formulated by modifying the AIN-93M diet according to Chen et al. (Chen et al., 2016) (Supplementary Table S1). HFD feeding was initiated at 8 weeks of age and continued for an additional 8 weeks, at which point the mice were fasted for 12 h euthanasia with ether. Blood was collected for serum biochemical analysis. The liver was quickly excised, entirely cleaned with ice-cold phosphate-buffered saline (PBS), weighed, and preserved in liquid nitrogen until use. The Ethics Committee approved all animal studies of Heilongjiang Bayi Agricultural University following the Chinese Guidelines for the Care and Use of Laboratory Animals.

### Histological Examination

A portion of liver tissue was fixed with 4% paraformaldehyde and embedded in paraffin. Rehydration was performed in a decreasing ethanol series, followed by staining with hematoxylin and eosin (H&E) (Ohtani et al., 2007). Frozen sections were prepared and stained with Oil Red O to determine hepatic lipid accumulation. The most severe hepatic inflammation in the representative histology sections was photographed using a microscope. Cells were fixed with 4% paraformaldehyde and stained with freshly diluted Oil Red O solution. Representative photomicrographs were captured using a system incorporated into the microscope.

### ELISA Assays

The tissues were placed in pre-cooled PBS and homogenized. Then the supernatant was recovered by centrifugation for analysis. The following substances were quantified by ELISA kits (Shanghai Lington Biotechnology Co., Ltd.). Liver injury was evaluated via concentrations of alanine aminotransferase (ALT) (Catalog No. BPE20168), aspartate aminotransferase (AST) (Catalog No. BPE20184); inflammation was assessed via concentrations of tumor necrosis factor-α (TNF-α) (Catalog No. BPE20220), interleukin-6 (IL-6) (Catalog No. BPE20012); oxidative stress was evaluated via malondialdehyde (MDA) (Catalog No. BPE20347), superoxide dismutase (SOD) (Catalog No. BPE20348); and lipid metabolism was determined via triglyceride (TG) (Catalog No. BPE20754), and total cholesterol (TC) (Catalog No. BPE20095). All assays were performed according to the manufacturers’ instructions.

### Cell Culture

Alpha mouse liver 12 (AML12) cells, a hepatocyte cell line from a mouse transgene for human transforming growth factor α, were kindly provided by the Stem Cell Bank, Chinese Academy of Sciences. The cells were cultured in the manufacturer’s recommended medium which was DMEM/F-12 (Dulbecco’s modified Eagle’s medium/Nutrient Mixture F-12, Gibco, 12400-024) medium containing 10% fetal bovine serum (FBS, CLARK, FB25015), 1% streptomycin (100 μg/ml) and penicillin-streptomycin (100 U/ml) (Solarbio, P1400), 1% transferrin (Gibco, 41400-045), and 40 ng/ml dexamethasone (Sigma, D4902-25MG). Cells were incubated with fresh medium at 37°C in 95% air and 5% CO_2_ and used in each experiment after 3 days.

### Cell Viability Analysis

The compound 3-(4,5-dimethyl-2-thiazolyl)-2,5-diphenyl-2-H-tetrazolium bromide (MTT) (Solarbio, M8180) was used to analyze cell viability. Cells were treated with different concentrations of HX for 20 h, and then 10 µl MTT was added for another 4 h. The culture medium was then completely removed, and dimethyl sulfoxide (DMSO) was added, followed by measurement of absorbance using a microplate reader. All MTT assays were performed at least three times in each group. Subsequently, results of the MTT assay were used to select five different concentrations of HX to add to the cell culture media. Real-time cell growth curves were measured using a real-time label-free cell analysis system (ACEA Biosciences).

### Cell Treatment

AML12 cells were seeded in 6-well plates. Hepatic steatosis *in vitro* was induced according to previously-established methods (Zhang et al., 2010). The AML12 cells were treated for 24 h with a mixture of free fatty acids (FFA) containing a 2:1 ratio of oleate (Sigma, O1383-5G) and palmitate (Sigma, P5585-10G), at a final FFA concentration of 1 mM. For the HX supplementation experiment, herbal extracts were added to the aforementioned medium containing 1 mM FFA for 24 h at high (25 mg/ml), medium (12.5 mg/ml), and low (6.25 mg/ml) concentrations.

### Protein Extraction and Western Blotting

Lysates of liver or AML12 cell samples containing protease inhibitors were prepared by adding frozen radioimmunoprecipitation assay (RIPA) buffer before determining the protein concentration with a bicinchoninic acid (BCA) kit (Beyotime, P0010). Protein samples (25 µg) were separated on 10% sodium dodecyl sulfate-polyacrylamide gel electrophoresis (SDS-PAGE) gels and then transferred onto polyvinylidene difluoride (PVDF) membranes. After blocking for 1 h in Tris-buffered saline (TBS) containing (0.1% Tween 20, pH 7.4 (TBST) with 5% nonfat milk, the membranes were incubated overnight with the indicated primary antibodies. After dilution with TBST (1:1,000 dilution), ACACA (Abcam, ab45174), FASN (CST, 3180S), SREBF1 (Novusbio, NB100-2215), NF-κB (CST, 6956S), p-NF-κB (CST, 3033S), IκBα (CST, 4814S), p-IκBα (CST, 2859S), IKKα (CST, 2682S), ATF6 (Abcam, ab203119), PERK (CST, 3192S), and IRE1 (Abcam, ab37073) monoclonal antibodies were used to detect the protein expression levels in the samples. Membranes were washed three times with TBST, followed by room temperature incubation for 30 min with horseradish peroxidase (HRP)-labeled goat anti-mouse or goat anti-rabbit (3:5,000; Beyotime, A0208, A0216) IgG secondary antibody in TBST plus 5% milk. The membranes were washed with PBS, developed using a HaiGene (M2301) detection kit, and then imaged with the AI600 imaging system. ImageJ software was used for protein quantitation.

### Confocal Laser Fluorescence Imaging

Cells were plated on coverslips at a density of 0.5 × 10^5^ cells per well, followed by treatment, and then fixed with 4% paraformaldehyde for 30 min (Kim et al., 2018). After incubation in blocking solution (3% bovine serum albumin (BSA), 5% goat serum, 0.5% Triton X-100 in PBS, pH 7.4) for 30 min, the cells were incubated overnight at 4°C with NF-κB (1:800) antibody. They were then washed with PBS five times and incubated with fluorescein isothiocyanate (FITC)-conjugated goat anti-mouse IgG (E1216, Santa Cruz Biotechnology) for 1 h. Hoechst 33342 (C1026, Beyotime) was used for nuclear staining. Fluorescence images were observed and photographed using an immunofluorescence microscope (Leica Microsystems).

### Flow Cytometry

An ROS Assay Kit (APPLYGEN, C1300) was used to measure ROS production according to the manufacturer’s instructions. Results were then analyzed by fluorescent microscopy and a flow cytometer (BD Biosciences, USA) (Cui et al., 2019b).

### Statistical Analysis

All analyses were performed using SPSS 22.0 (SPSS, Inc., Chicago, IL, USA) statistical software.

*In vivo* experiments, 6 mice were treated in each group, one-way ANOVA analyzed data, and multiple comparisons were employed, and results are expressed as the mean ± SEM (*n* = 6 per group). *p* < 0.05 was considered significant between any two groups by analysis of variance (ANOVA) followed by Tukey’s post hoc test.

*In vitro* experiments, three concentrations of HX were added to cell culture, statistical analyses were performed using Student’s t-test, and continuous data are presented as the mean with S.E.M. Multiple comparisons were performed using one-way analysis of variance (ANOVA) followed by Tukey’s post hoc test. All tests were two-sided, and *p* < 0.05 was considered significant.

## Results

### Composition of Compounds in HX

**FIGURE 1:**
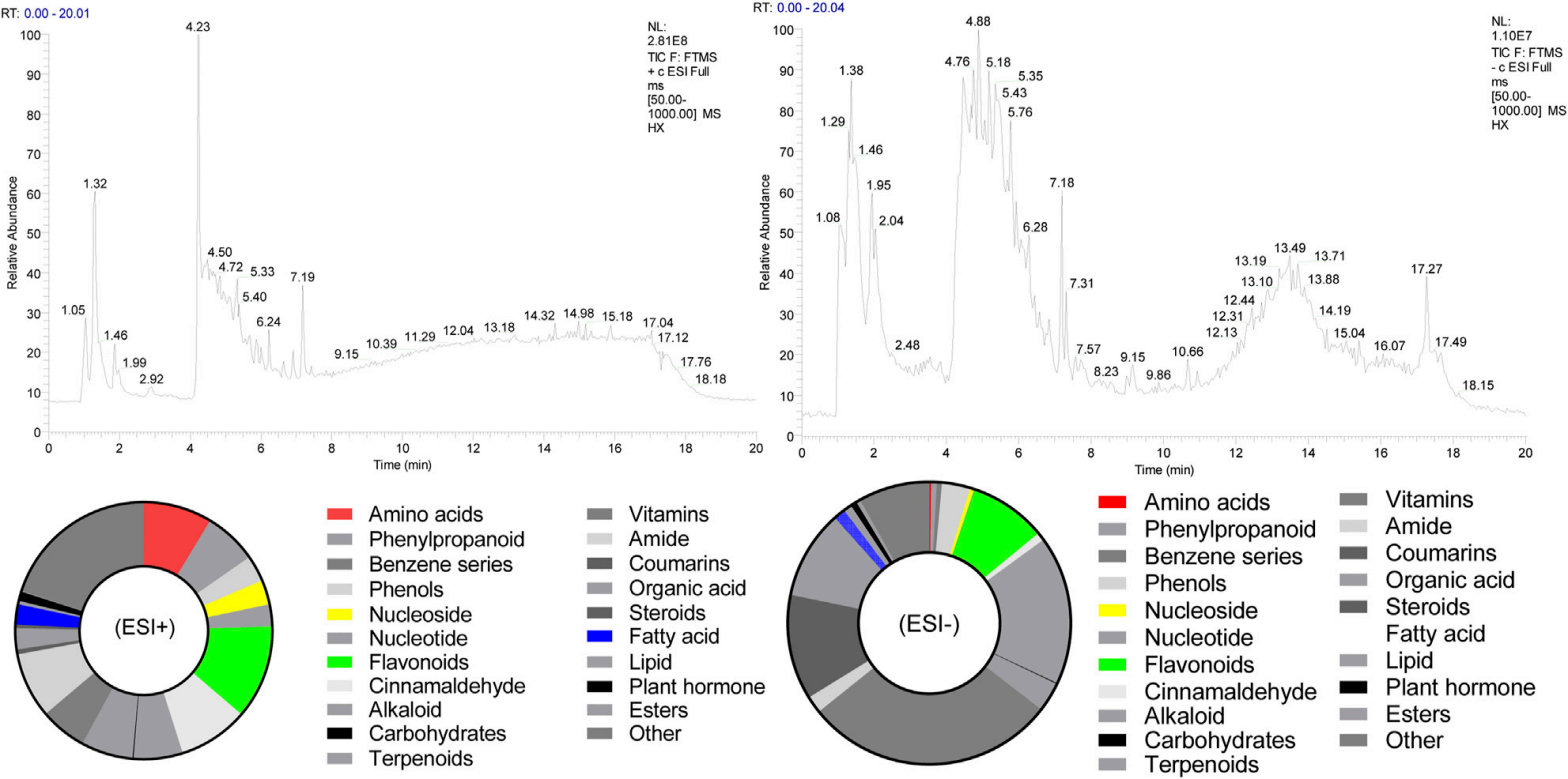
Total ion chromatogram of HX. (ESI+) represents the positive ion detection mode, in which the mass analyzer scans only positively charged ions and filters out negatively charged ions. This allows us to obtain positively charged ion information during the detection process; (ESI-) denotes the negative ion detection mode, in which the mass analyzer scans only negatively charged ions and filters out positively charged ions, thus obtaining information regarding negatively charged ions.

The chemical composition of HX (peak MS spectra) is reported in Figure 1. Feature extraction was performed on the data and preprocessed with XCMS in R software. Data were then normalized and edited into a two-dimensional data matrix in Excel 2010 software, including retention time (RT), mass-to-charge ratio (MZ), observations (samples), and peak intensity. Concentrations of identified substances are reported in Supplementary Tables S2, S3. Ninety-nine compounds were identified, with 33% of those classified as flavonoids. The full spectrum of constituents was identified based on the METLIN database (https://metlin.scripps.edu).

### Active Ingredient Targets and Disease Targets

**FIGURE 2:**
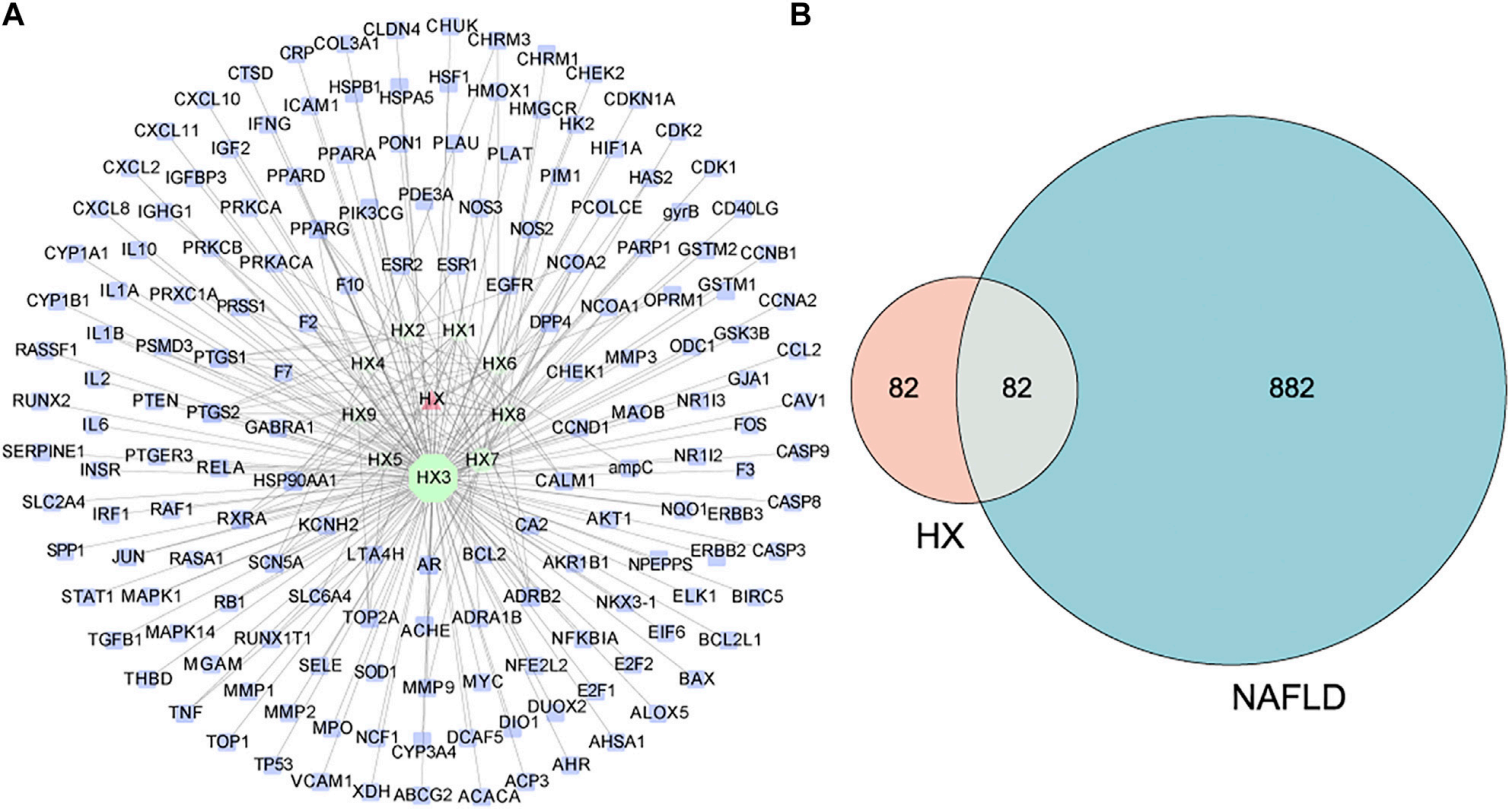
Predicted effects of HX against NAFLD. **(A)** The compound target network of HX, which consists of 164 compound target nodes, 174 compound nodes, and 239 edges. The hexagons in green represent compounds, while the squares in blue denote potential targets. The node size of gene targets is proportional to the number of degrees. Lines represent the relationship between compounds and target nodes. **(B)** Venn diagram of active ingredient targets and disease proteins.

Evaluation of the Drugbank and TCMSP databases revealed 164 potential targets from 9 bioactive compounds. Figure 2A depicts 174 nodes (9 bioactive compounds and 164 potential targets) and 239 edges. The red triangles denote HX, the green hexagons denote bioactive compounds, the blue rectangles denote potential targets, and each edge indicates an interaction among them. Green nodes represent compounds, and the degree value determines the size of the nodes. The potential active compounds were 5-hydroxy-7,4′-dimethoxyflavanon, quercetin 7-O-β-D-glucoside, quercetin, diop, acanthoside B, phenanthrone, irisolidone, genkwanin, and 3,23-dihydroxy-12-oleanen-28-oic acid (Supplementary Table S4). After translation, 964 target genes of disease were retrieved from potential targets in the protein. Targets associated with NAFLD were identified in the DisGeNET databases.

### Target Network Analysis

**FIGURE 3:**
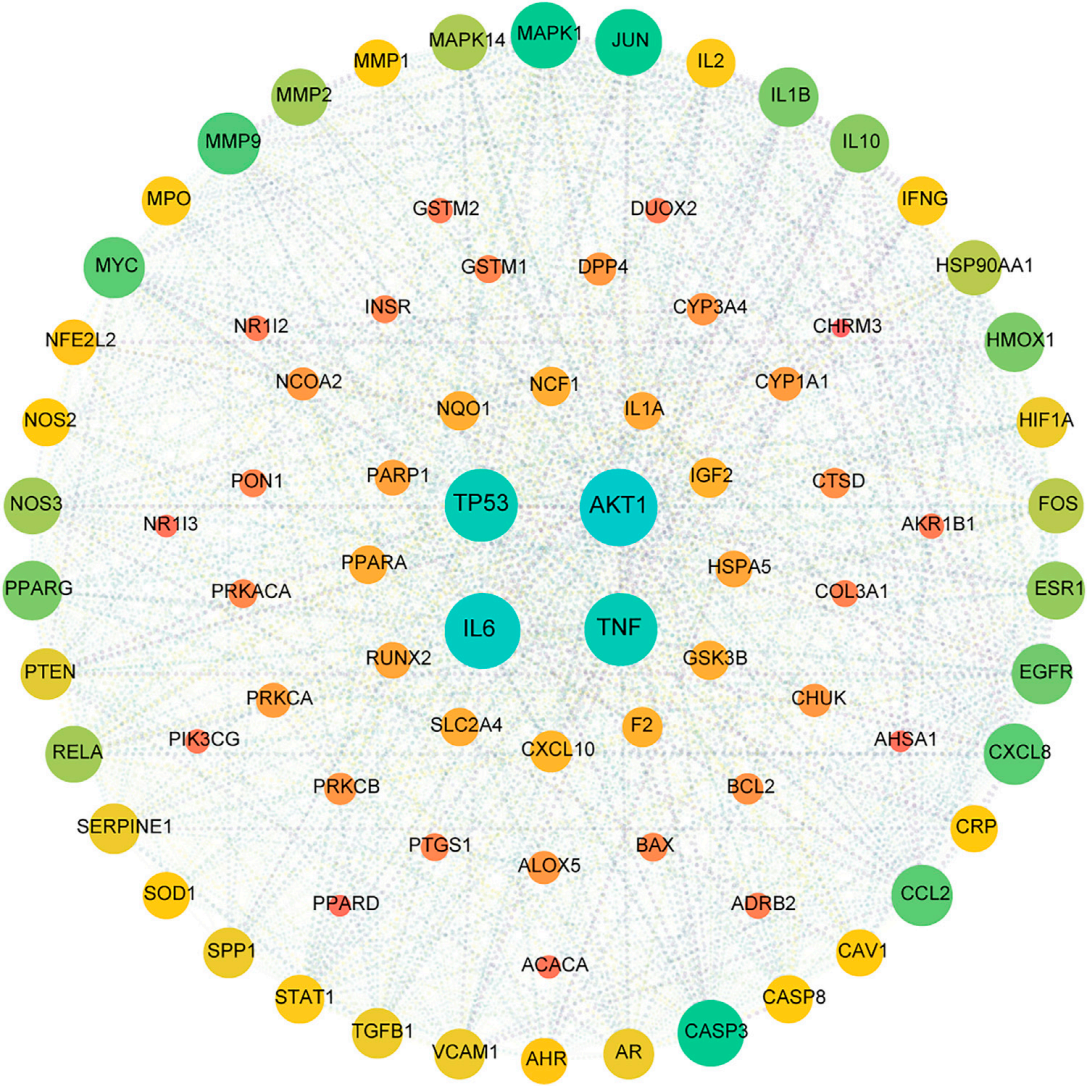
PPI network for HX acting on NAFLD. This target network was composed of 82 nodes and 1,246 edges. A node represents one target, the degree value determines the node’s size, and the edges represent protein–protein relationships. The 82 nodes represent therapeutic targets for HX acting on NAFLD, and the lines denote the relationship of 1,246 targets. The nodes (TP53, AKT1, IL6, and TNF) have higher degrees.

All active compound target proteins and disease-associated proteins were divided into two independent groups. The sets and their relationships were represented in a closed format with fixed positions, yielding a Venn diagram and 82 interacting proteins (Figure 2B). The Cytoscape plug-in generated protein-protein interaction (PPI) networks based on the STRING database and topological data analysis to obtain a PPI network of 82 essential targets in Figure 3. Through network prediction, it was revealed that *Tnf, Il6, Tp53, and Akt1* were essential target genes in the first few degrees.

### Pathway Enrichment Analysis of HX Target Proteins

**FIGURE 4:**
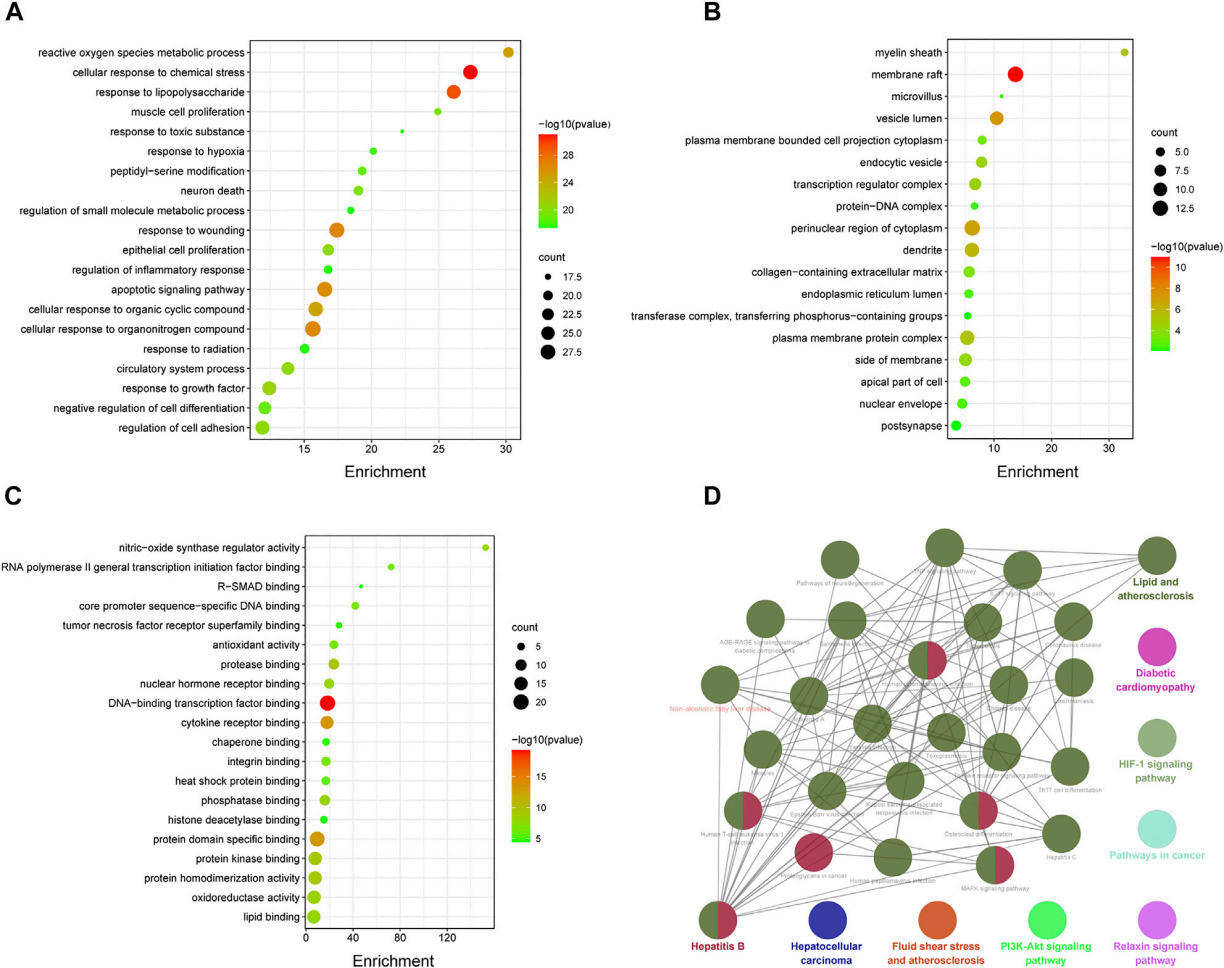
Enrichment analysis of potential genes involved in the anti-NAFLD effects of HX. **(A)** Part of a GO functional analysis—biological process. **(B)** Part of a GO functional analysis—cellular components. **(C)** Part of a GO functional analysis—molecular functions. **(D)** Enriched KEGG pathways of potential targets of HX.

Eighty-two common targets were studied using GO and pathway enrichment analysis functions of the Metascape platform; an advanced bubble chart was generated with the top 20 results selected according to *p*-value (*p* < 0.01). The analysis included BP, CC, and MF (Figures 4A–C). BP analysis showed that HX acts mainly through cellular responses to chemical stress and lipopolysaccharide. The CC was mostly composed by vesicle lumen, perinuclear region of the cytoplasm, and transferase complex. Results of the MF analysis showed that HX exerted its function mainly through interactions with DNA-binding transcription factor binding, cytokine receptor binding, oxidoreductase activity, and antioxidant activity. On these basis, KEGG analyses were performed to elucidate potential targets in NAFLD of the components that make up HX. As shown in Figure 4D, HX mainly functioned in Fluid shear stress and atherosclerosis, Hepatocellular carcinoma, and Diabetic cardiomyopathy (ClueGo analysis). The common parts of the KEGG enrichment pathways were further extracted, and Lipid and atherosclerosis, Nonalcoholic fatty liver disease, TNF signaling pathway, and MAPK signaling pathway were the main common factors (Supplementary Table S5).

### HX Alleviated Liver Lesions Induced by High-Fat Diet

To exclude the effect of HX on cell viability, tests were carried out 20 h after treatment of AML12 cells with different concentrations of HX. When cells were treated with 30, 60, and 90 mg/ml HX for 20 h, the inhibitory rate on cell viability was 110.1, 97.9, and 92.9%, respectively, and the IC_50_ value for AML12 cells was 199.3 mg/ml (Supplementary Figure S1). According to the experimental results, a concentration of HX was selected from the cell experiments for use in the follow-up experiment because it would provide sufficient safety.

**FIGURE 5:**
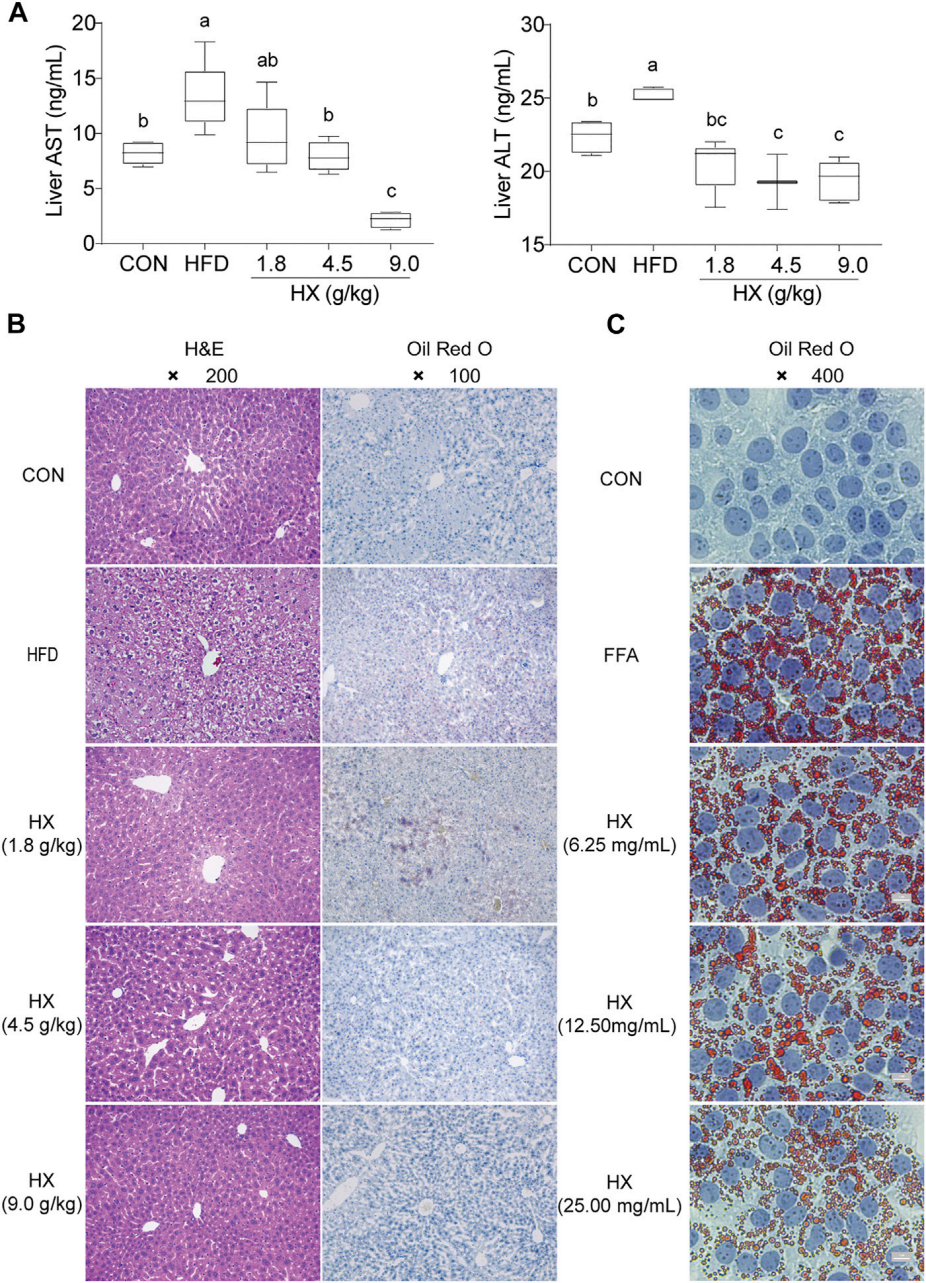
**(A)** Concentrations of ALT and AST **(B)** H&E and Oil-Red-O staining in the liver tissue of mice after 8 weeks of HFD feeding, and **(C)** Oil-Red-O staining in AML12 cells incubated without or with FFA and different concentrations of HX (6.25, 12.50, and 25.00 mg/ml). A one-way ANOVA was used to analyze data, and multiple comparisons were employed, and the results are expressed as the mean ± SEM (*n* = 6 per group). Statistical analyses were performed using Student’s t-test. Different lowercase letters a, b and c denote significant differences between any two groups (*p* < 0.05), but with the same lowercase letters, like bc and c, ab and a or b means no differences between the two groups (*p* > 0.05). ALT, alanine aminotransferase; AST, aspartate aminotransferase; CON, control; HFD, high-fat diet; FFA, free fatty acid; HX, Huo Xiang.

Compared with the Con group, the content of ALT and AST in the liver homogenate of the HFD group were significantly greater (*p* < 0.01). Compared with the HFD group, the amounts of ALT and AST in the HX groups were lower with increasing concentrations of HX in the diet (Figure 5A).

Hepatocytes were structurally intact in the Con group, with polygonal shape, clear borders, red cytoplasm, no vacuoles, centered nuclei, clear outlines of the hepatic lobules, and stable structure. The hepatic cords were radially arranged with the central vein as the axis. However, the HFD group exhibited diffuse vacuoles around the central vein and portal vein, unclear borders between cells in the visual field, and swollen or inflated hepatocytes. Numerous sub-circular vacuoles were visible inside the cells. The vacuoles squeezed the nuclei of hepatocytes to one side, compressed and narrowed the hepatic sinusoids, and the structures of hepatic sinusoids and hepatic cords were unclear.

Compared with the HFD group, there was greater normality in liver tissue from the HX groups in terms of sinuses, the structural arrangement of hepatic cords, the morphology of liver cells and fat globules, and balloon-like changes (Figure 5B).

Oil Red O staining of the liver tissue showed that the nuclei in the Con group were blue, there were no apparent orange lipid droplets, the space between liver cells was clear, and the structure of the liver sinuses was typical. In the HFD group, however, hepatocytes were enlarged, with diffused lipid droplets in the field of vision. In the HX groups, decreasing amounts of orange lipid droplets were evident, denoting decreased lipid accumulation (Figure 5C).

Oil Red O staining revealed many lipid droplets in the AML12 cells in the FFA group, and there were chain and mass changes in some of the fusions. In the low-, medium-, and high-dose HX groups, the number of orange lipid droplets in the hepatocytes decreased as the HX dose increased, and the degree of lipid accumulation was reduced, which was consistent with the changes observed in the liver tissue (Figure 5C).

### HX Increased the Liver Antioxidant Capacity During Nonalcoholic Fatty Liver Disease Induced by High-Fat Diet

**FIGURE 6:**
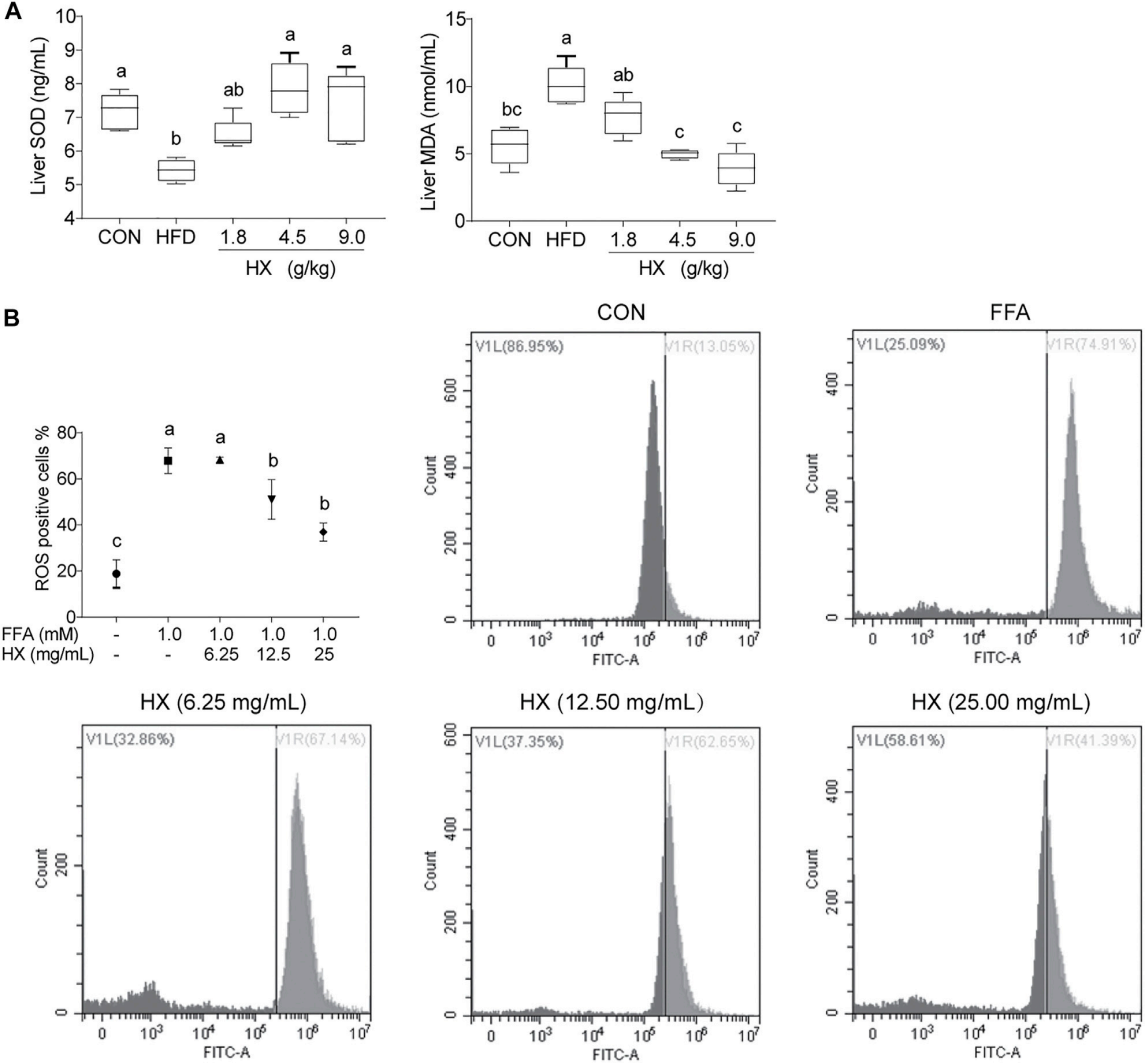
**(A)** Concentrations of SOD and MDA in liver tissue of mice after 8 weeks of HFD feeding and **(B)** ROS percentage in AML12 cells incubated without or with FFA and different concentrations of HX (6.25, 12.50, and 25.00 mg/ml). “Count”: cell counts; VL: Visible left of ROS negative percentage dye in cells; VR: Visible right of ROS positive percentage dye in cells. A one-way ANOVA was used to analyze the data for A, and multiple comparisons were employed, and the results are expressed as the mean ± SEM (*n* = 6 per group). Statistical analyses were performed using Student’s *t* test. Different lowercase letters a, b and c denote significant differences between any two groups (*p* < 0.05), but with the same lowercase letters, like bc and c, ab and a or b means no differences between the two groups (*p* > 0.05). SOD, superoxide dismutase; MDA, malondialdehyde. CON, control; FFA, free fatty acid; HX, Huo Xiang.

Compared with the Con group, the activity of SOD in the liver of the HFD group was significantly lower (*p* < 0.01), while the concentration of MDA was significantly greater (*p* < 0.01). Furthermore, HX significantly decreased the MDA level (*p* < 0.01) in a dose-dependent manner (Figure 6A). In AML12 cells, the amount of ROS in the FFA group was significantly greater (*p* < 0.01) compared with the Con group, while the amount of ROS in the HX groups was significantly lower (*p* < 0.01) compared with the Con group (Figure 6B).

### HX Suppressed Inflammation in the Liver and AML12 Cells

**FIGURE 7:**
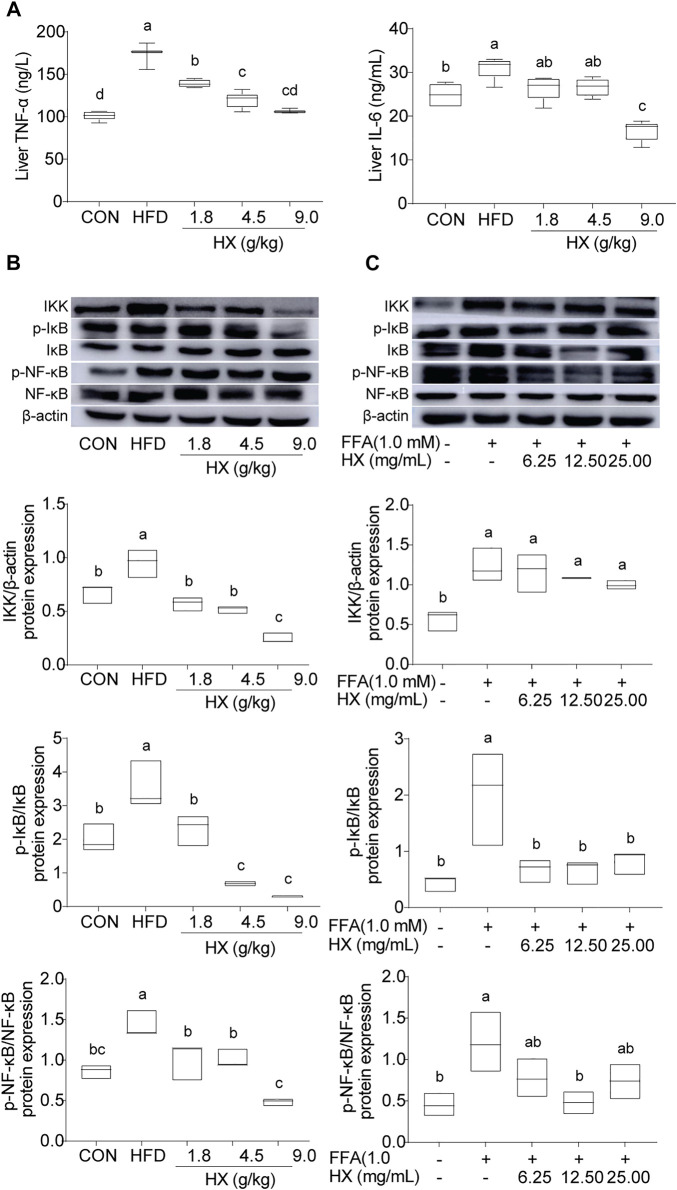
**(A)** Concentrations of TNF-α and IL-6 and **(B)** components of the inflammatory pathway in the liver tissue of mice after 8 weeks of HFD feeding, **(C)** and in AML12 cells incubated without or with FFA and different concentrations of HX (6.25, 12.50, and 25.00 mg/ml). A one-way ANOVA was used, and multiple comparisons were employed, and the results are expressed as the mean ± SEM (*n* = 6 per group). Statistical analyses were performed using Student’s *t* test. Different lowercase letters denote significant differences among groups (*p* < 0.05). TNF-α, tumor necrosis factor-α; IL-6, interleukin-6; IKK, inhibitor of κB kinase; NF-κB, nuclear factor-ĸB; IκB, inhibitor of NF-κB; p-IκB, phosphorylation inhibitor of NF-κB; p-NF-κB, phosphorylation nuclear factor-ĸB; CON, control; HFD, high-fat diet; HX, Huo Xiang.

**FIGURE 8:**
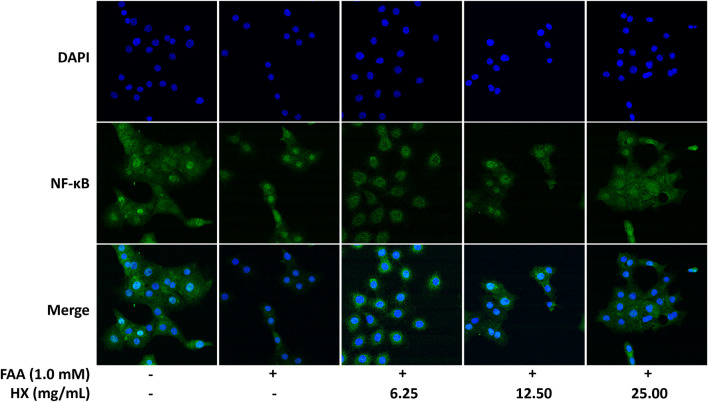
Immunofluorescence images demonstrating nuclear staining **(DAPI, blue; top images)**, NF-κB droplet staining **(green; middle images)**, and an overlay of both **(merge, bottom images)** in AML12 cells incubated without or with FFA and different concentrations of HX (6.25, 12.50, and 25.00 mg/ml). FFA, free fatty acid; HX, Huo Xiang.

Compared with the Con group, the levels of TNF-α and IL-6 (Figure 7A) and abundance of IKK, p-NF-κB/NF-κB, and p-IκB/IκB protein in the liver of the HFD group were significantly increased (*p* < 0.01), and were significantly lower (*p* < 0.01) in the 9.0 g/kg HX groups (Figure 7B). Furthermore, HX significantly decreased p-NF-κB/NF-κB and p-IκB/IκB abundance in the AML12 cells (*p* < 0.01) (Figure 7C). In AML12 cells, compared with the Con group, NF-κB protein was mainly distributed in the nucleus of the FFA group, while the abundance of NF-κB protein was more significant in the cytoplasm of the high-dose HX groups (Figure 8).

### HX Reduced Fat Deposition During NAFLD Induced by a High-Fat Diet

Compared with the Con group, the amounts of TC and TG in the liver homogenate of the HFD group were significantly greater (*p* < 0.01). Compared with that of the HFD group, the amounts of TC and TG in the liver homogenate of the HX groups were significantly lower (*p* < 0.01) (Supplementary Figure S2A). Compared with the Con group, the abundance of SREBF1, ACACA, and FASN was significantly upregulated in the HFD group. Compared with the HFD group, there was a significantly lower abundance of SREBF1, ACACA, and FASN in the HX groups (Supplementary Figure S2B). In addition, the protein abundance of SREBF1 and FASN decreased in a dose-dependent manner in the HX groups. Furthermore, the abundance trends of SREBF1, ACACA, and FASN in AML12 cells were consistent with those in liver tissue (Supplementary Figure S2C).

### HX Alleviated Endoplasmic Reticulum Stress During Nonalcoholic Fatty Liver Disease Induced by a High-Fat Diet

Compared with the standard group, protein abundance of PERK, IRE1, and ATF6 in the livers of the HFD group was significantly increased (*p* < 0.05), although no difference was observed in the groups with different HX dosage (*p* > 0.05). In contrast, marked decreases in the abundance of PERK, IRE1, and ATF6 were observed in the HX groups compared with the HFD group (*p* < 0.05) (Supplementary Figure S3A). Compared with the Con cells, cells treated with FFA exhibited significantly greater amounts of PERK and IRE1 (*p* < 0.05). Compared with the FFA-treated cells, there was a considerably lowered abundance of PERK and IRE1 proteins (*p* < 0.05) in a dose-dependent manner in cells pretreated with HX (Supplementary Figure S3B).

## Discussion

Based on traditional studies following the “one drug for one target” concept, in 2007 Hopkins proposed the idea of network pharmacology (Hopkins, 2007), which is a comprehensive discipline integrating systems biology, information networks, computer science, and pharmacology. Specifically, a single drug regulates multiple proteins simultaneously, or multiple drugs act together on one protein to produce better therapeutic effects on diseases.

Network pharmacology has become a popular tool for research on the traditional Chinese medicines’ pharmacological basis and mechanisms of action (TCM). This approach allows for modeling and studying biological systems using complex networks. It is widely used in the screening of active components in Chinese medicines, repositioning of drugs, exploration of the mechanism of compatibility of Chinese medicines, and explanation of the mechanism across multiple components, targets and pathways (Zheng et al., 2020; Zhou et al., 2021). According to literature reports, we predicted the relationship between the effective target of HX and NAFLD disease-related proteins through network pharmacology, which allowed us to speculate about the mechanism of HX action on NAFLD. The effect of HX on NAFLD was further verified through *in vivo* and *in vitro* experiments.

In this study, we used network pharmacological tool and discovered that HX has the ability to regulate molecules. These molecules are targets for the treatment of NAFLD, and are found in the signaling pathway for NAFLD (Chyau et al., 2020). The main compounds contained in HX that are effective for the treatment of NAFLD are flavonoids. The results of the topological analysis showed that quercetin attained the most targets, and these components were highly correlated with the core targets predicted by NAFLD. Quercetin can reduce hepatic stellate cell activation and prevent hepatic fibrosis by inhibiting the TGF-β1/Smads signaling pathway, with activation of PI3K/Akt signaling inhibiting autophagy caused by liver fibrosis and reduce liver injury (Wu et al., 2017). PPI network topology analysis showed that the inflammation related key targets, TNF, and IL-6 were essential components for the effect of HX on NAFLD. Hepatic steatosis and inflammation pathogenesis accour through the secretion of cytokines such as TNF- α and IL-6 (Zhang et al., 2019), which are critical pro-inflammatory factors in the development of steatohepatitis (Jing et al., 2015, 3). Pathway and function enrichment analysis also showed that HX mainly regulates metabolism-related pathways. Hepatic accumulation of lipids is the hallmark of NAFLD and subsequently leads to cellular stress, inflammation, and hepatic injury, eventually resulting in chronic liver disease (Spahis et al., 2017; Dalekos et al., 2020). Abnormal lipid accumulation is associated with perturbed hepatocyte endoplasmic reticulum (ER) proteostasis in steatotic livers (Kitade et al., 2017).

In addition to flavonoids, the other main components of HX are terpenoids, ketones, alcohols, and aldehydes (Yamani et al., 2014). According to the mass value in the detected data, the entire spectrum identification was carried out based on the network database Metlin. The identified substances mainly included flavonoids, caffeic acid, ellagic acid, and other phenolic substances. Fatty acids such as stearic acid and palmitic acid, organic acids such as fumaric acid, and NADH, coenzyme Q, and other coenzymes were identified by the full spectrum. (see Supplementary Tables S2, S3). Pengran (Cao et al., 2017) and He Qin (Li HQ. et al., 2013) also analyzed the chemical composition of HX and reported that its main components acacetin and tilianin exhibited significant anticoagulant activity. However, it is well known that flavonoids elicit many pharmacological effects, including antioxidant, anti-inflammatory, analgesic, immunomodulatory, antiaging, hypolipidemic, and anti-tumor effects (Lei et al., 2019). Although the present study did not attempt to isolate a unique compound in HX, but instead used an extract of the whole plant, the results demonstrated that the mixture of compounds had the capability to reduce lipid accumulation, enhance antioxidant capacity, and reduce inflammation.

Oxidative stress is a critical factor of the “second hit” theory during NAFLD (Borrelli et al., 2018). Accumulation of lipid in hepatocytes leads to acceleration of mitochondrial β-oxidation capacity, which enhances the production of ROS that often exceeds the antioxidant capacity of the liver and causes oxidative stress (Hwang et al., 2020). Previous results showed that the activity of SOD in serum decreased and MDA significantly increased in patients with NAFLD. (Świderska et al., 2019). The antioxidant effect of HX is dose-dependent, leads to increased HO-1 protein and enzymatic activity, and protects cells from H_2_O_2_-induced cytotoxicity (Oh et al., 2006). This response also attenuates UVB-induced photoaging by upregulating antioxidant enzymes (Oh et al., 2016) and reducing ROS production (Shin et al., 2018). Our results showed that compared with the HFD group, the activity of SOD in the livers of the HX groups significantly increased, while activity of MDA and ROS in hepatocytes decreased. Thus, we speculate that HX directly decreases lipid peroxidation by increasing antioxidant enzymatic activity.

An increase in mitochondrial β-oxidation induced by feeding HFD can cause oxidative stress, and an increase in ROS production can activate the inflammatory pathway regulated by IKK/NF-κB (de Meneses Fujii et al., 2014). As an inflammatory signaling pathway component, NF-κB induces the transcription of numerous pro-inflammatory cytokines (TNF-α and IL-6) (Cortez et al., 2013, 6). In terms of anti-inflammatory effects, the essential oils in HX suppressed nitric oxide (NO) production by inhibiting NF-κB in lipopolysaccharide (LPS)-stimulated RAW264.7 cells (Lee et al., 2012, 3). Supplemental HX prevented NF-κB’s activation and translocation to the nucleus (Hong et al., 2001; Oh et al., 2005). In the present study, feeding HX decreased the NF-κB-induced transcription of inflammatory cytokines in HFD-induced NAFLD and inhibited the protein abundance of IKK-NF-κB signaling pathway components. These results suggest that HX might inhibit activation of IKK/IKB/NF-κB signaling to interrupt the inflammatory cascade and thereby reduce the “second hit” of inflammatory factors on the liver.

Serum enzymology and blood lipids are stock indices used for the clinical diagnosis of NAFLD, but liver histology remains the “gold standard” (Castera et al., 2019). Our results showed that compared with the HFD group, there were decreased amounts of TC and TG and lipid accumulation in the hepatocytes of the HX groups. HFDs can lead to hyperlipidemia and disorders of liver lipid metabolism, including accumulation of TG and liver cell degeneration. According to the pathogenesis of HFD-induced NAFLD, many natural products can regulate hepatic lipogenesis and esterification of fatty acids into TG (Yh et al., 2020). SREBF1 is a crucial regulator of lipogenesis, and overactivation of this regulatory process is one characteristic of NAFLD (Khaleel et al., 2018). Hence, the downregulation of ACACA and FASN could prevent NAFLD (Cui et al., 2017). Recent studies have reported that ethanol extracts from HX conferred anti-adipogenicity effects in 3T3-L1 adipocytes (Park et al., 2016)*.* Pogostone from HX reduced serum TNF-α and IL-6, increased IL-10, and increased the level of nonprotein sulfhydryl in gastric tissue to strengthen the inflammatory response (Chen et al., 2015). An anti-atherogenic effect was observed when HX was administered to low-density lipoprotein receptor^−/−^ mice. Tilianin, a major component of HX, inhibits the TNF-α-induced expression of VCAM1 in cultured human umbilical vein endothelial cells (HUVECs) (Hong et al., 2001). Thus, our finding that HX decreased the abundance of SREBF1, ACACA, and FASN to modulate lipid accumulation and attenuate NAFLD induced by a HFD suggests a potent anti-lipogenic effect.

ER stress is a crucial feature of NAFLD (Ding et al., 2017). The ER is the central organelle where protein folding and post-translational modifications of proteins occur, and it acts as a significant intracellular calcium reservoir in the cell. A HFD activates ER stress in the liver, with PERK, IRE1, and ATF6 also being involved in the development of NAFLD (Cao et al., 2012). The unfolded protein response acts as a complementary adaptive machinery against ER stress. Three ER transmembrane receptor proteins coordinate it: inositol-requiring kinase 1 (IRE1), double-stranded RNA-activated protein kinase (PKR)-like endoplasmic reticulum kinase (PERK), and activating transcription factor 6 (ATF6) (Wang et al., 2018). We observed that HX alleviated hepatic steatosis by decreasing ER stress-related protein expression *in vivo* and *in vitro*, which confirmed the results of Sandoval and Carrasco (Sandoval and Carrasco, 1997), who concluded that HX could successfully prevent ER stress.

**FIGURE 9:**
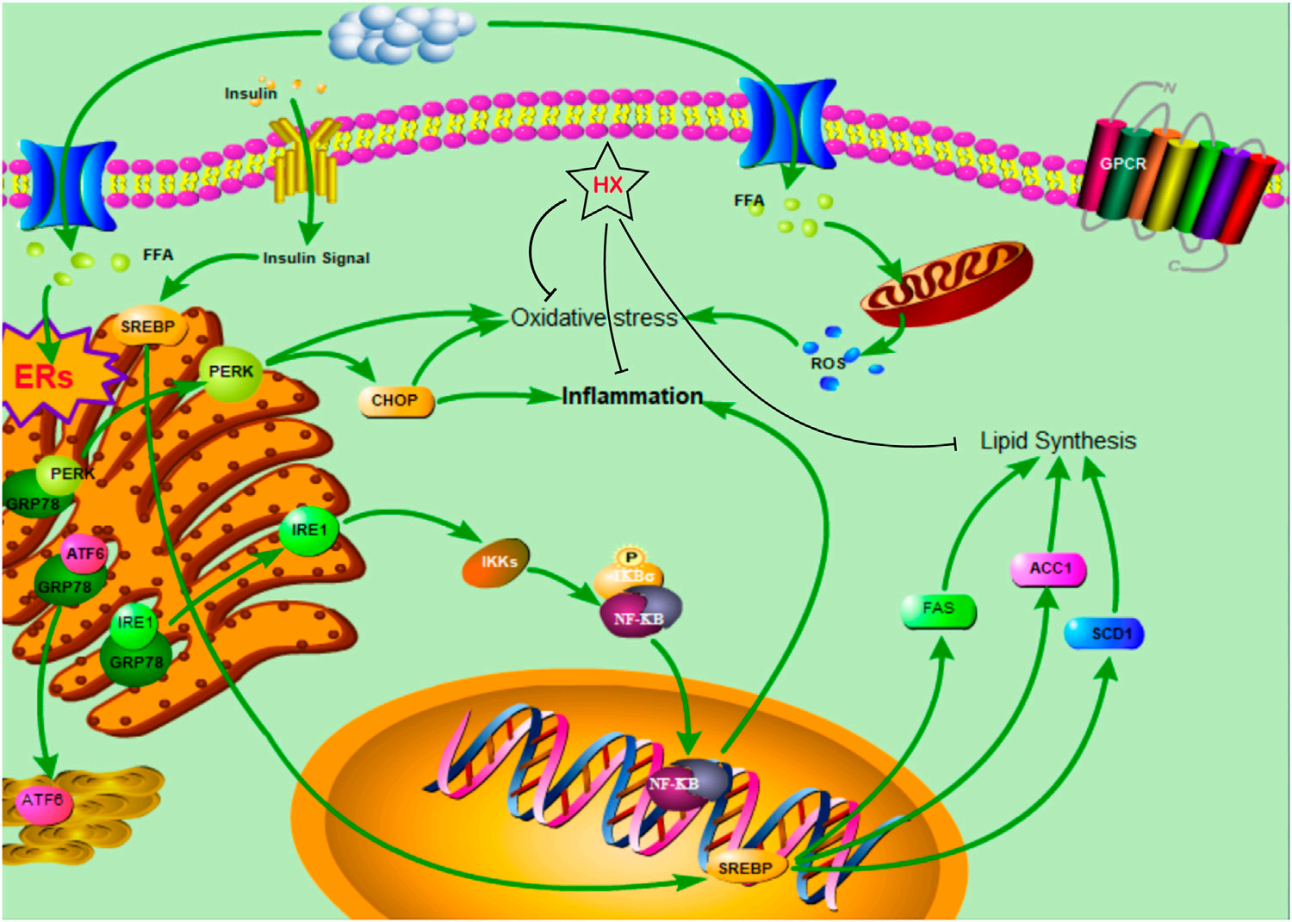
The protective mechanism of a water extract of Huo Xiang (HX) on free fatty acid (FFA)-induced nonalcoholic fatty liver disease (NAFLD).

HX treatment significantly reduced FFA-induced disruption of lipid metabolism, oxidative stress, and inflammatory responses in AML12 cells and inhibited lipid synthesis in the SREBF1/FASN/ACACA pathway, thereby ameliorating NAFLD symptoms and protecting the liver (Figure 9). These results are consistent with our predicted targets and signaling network analysis.

## Conclusion

A preliminary prediction of the potential active compounds, core targets and possible mechanisms of HX action for the treatment of NAFLD were generated. The network pharmacological method provided new avenues for future development and research of HX in the context of health disorders. HX inhibits oxidative stress and inflammatory responses through various signaling pathways and reduces lipid metabolism disorders. Among the components in HX, quercetin may be particularly beneficial for treatment of NAFLD, which is a multifactorial and complex condition including lipid accumulation in hepatocytes. Further studies are needed to determine the regulatory role and mechanisms whereby HX elicits beneficial effects in animal models of NAFLD.

## Data Availability

The datasets presented in this study can be found in online repositories. The names of the repository/repositories and accession number(s) can be found below: https://www.jianguoyun.com/p/DauG-wcQoOvyCRiZpJEE.

## Abbreviations

ACACA, acetyl-CoA carboxylase-alpha; ALT, alanine aminotransferase; AML12, alpha mouse liver 12; AST, aspartate aminotransferase; ATF6, activating transcription factor 6; FASN, fatty acid synthase; FFA, free fatty acid; HX, Huo Xiang; LC/MS, liquid chromatography-electrospray ionization/mass spectrometry; H&E, hematoxylin and eosin; HFD, high-fat diet; IL-6, interleukin-6; IKK, inhibitor of κB kinase; IκB, inhibitor of NF-κB; IR, insulin resistance; IRE1, inositol-requiring enzyme-1; MDA, malondialdehyde; MTT, 3-(4,5-dimethyl-2-thiazolyl)-2,5-diphenyl-2-H-tetrazolium bromide; NAFLD, Nonalcoholic fatty liver disease; NF-ĸB, nuclear factor-ĸB; PERK, protein kinase (PKR)-like endoplasmic reticulum kinase; SOD, superoxide dismutase; SREBF1, sterol regulatory element binding transcription factor 1; TC, total cholesterol; TCM, traditional Chinese medicine; TG, triglyceride; TNF-α, tumor necrosis factor-α.
