# Supplementary material for: Network Pharmacology-Based Analysis of Pogostemon cablin (Blanco) Benth Beneficial Effects to Alleviate Nonalcoholic Fatty Liver Disease in Mice
Source: Front Pharmacol. 2021 Nov 24;12:789430. doi: 10.3389/fphar.2021.789430 (PMC8652055; doi:10.3389/fphar.2021.789430)
Supplement: Supplementary file 1 [file DataSheet1.docx]

**Supplementary data**

**Supplementary Figure**


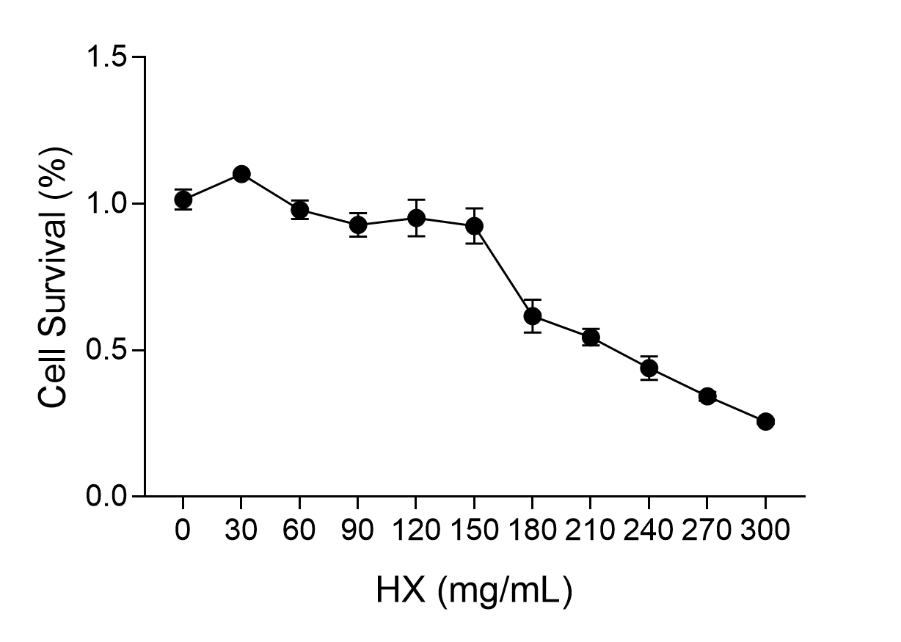


**Supplementary Figure S1.** Effect of HX on AML12 cell viability in response to different concentrations of HX after incubation for 20 h. Data represent mean ± SEM of three independent experiments.


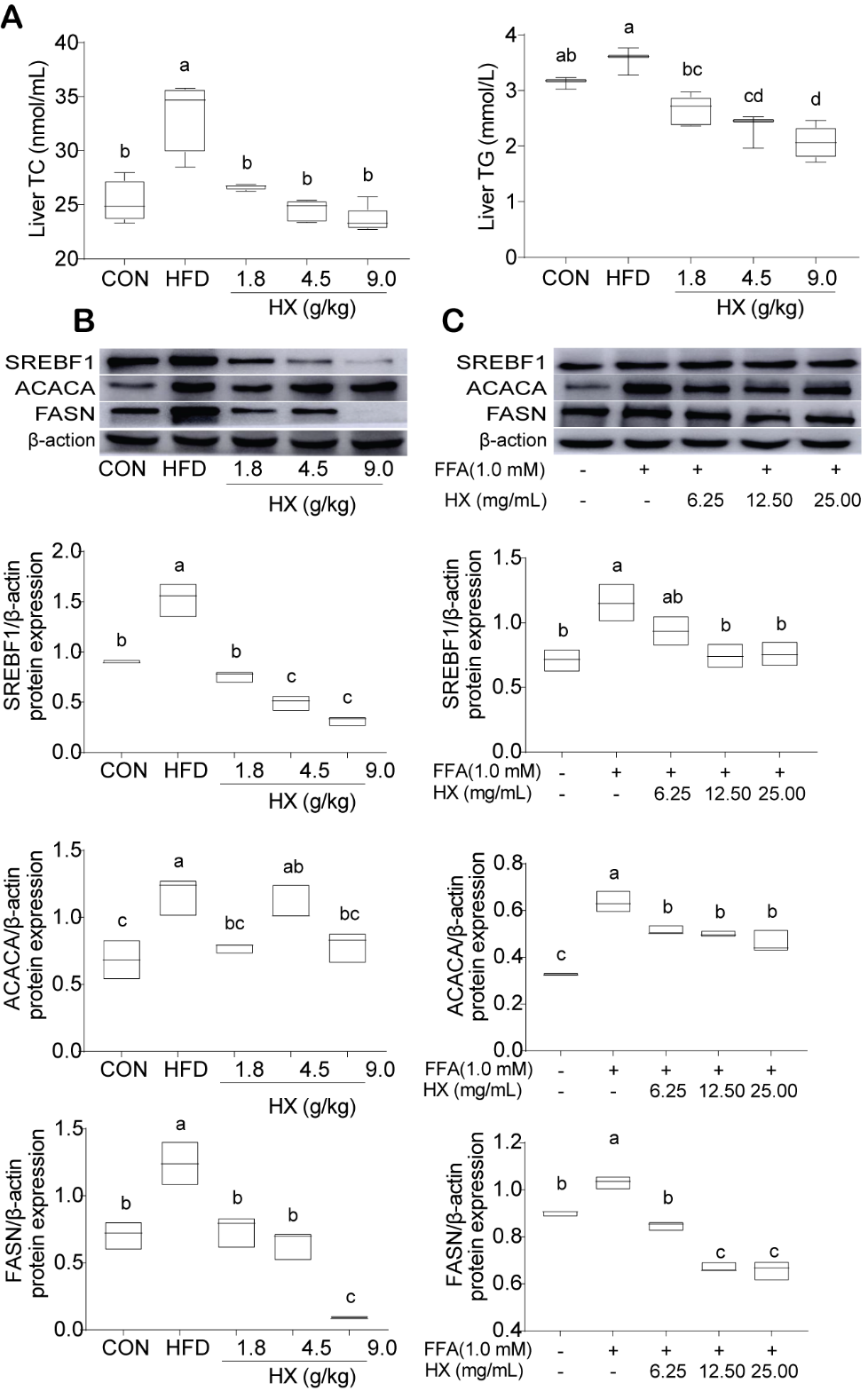


**Supplementary Figure S2.** Total cholesterol (TC) and triglyceride (TG) (panel A), and analysis of lipid metabolism-related proteins in liver (panel B) of mice after 8 weeks of HFD feeding and AML12 cells incubated without or with FFA and different concentrations of HX (6.25, 12.50, and 25.00 mg/mL) (panel C). Data were analyzed by a one-way ANOVA and multiple comparisons was employed, and results are expressed as the mean ± SEM (n=6 per group). Statistical analyses were performed using Student’s *t* test. Different lowercase letters a, b and c denote significant differences between any two groups (*P* < 0.05), but with the same lowercase letters, like bc and c, ab and a or b, ab and bc means no differences between the two groups (*P* > 0.05). SREBF1, sterol regulatory element binding transcription factor 1; ACACA, acetyl-CoA carboxylase; FASN, fatty acid synthase; CON, control; HFD, high-fat diet; FFA, free fatty acid; HX, Huo Xiang.


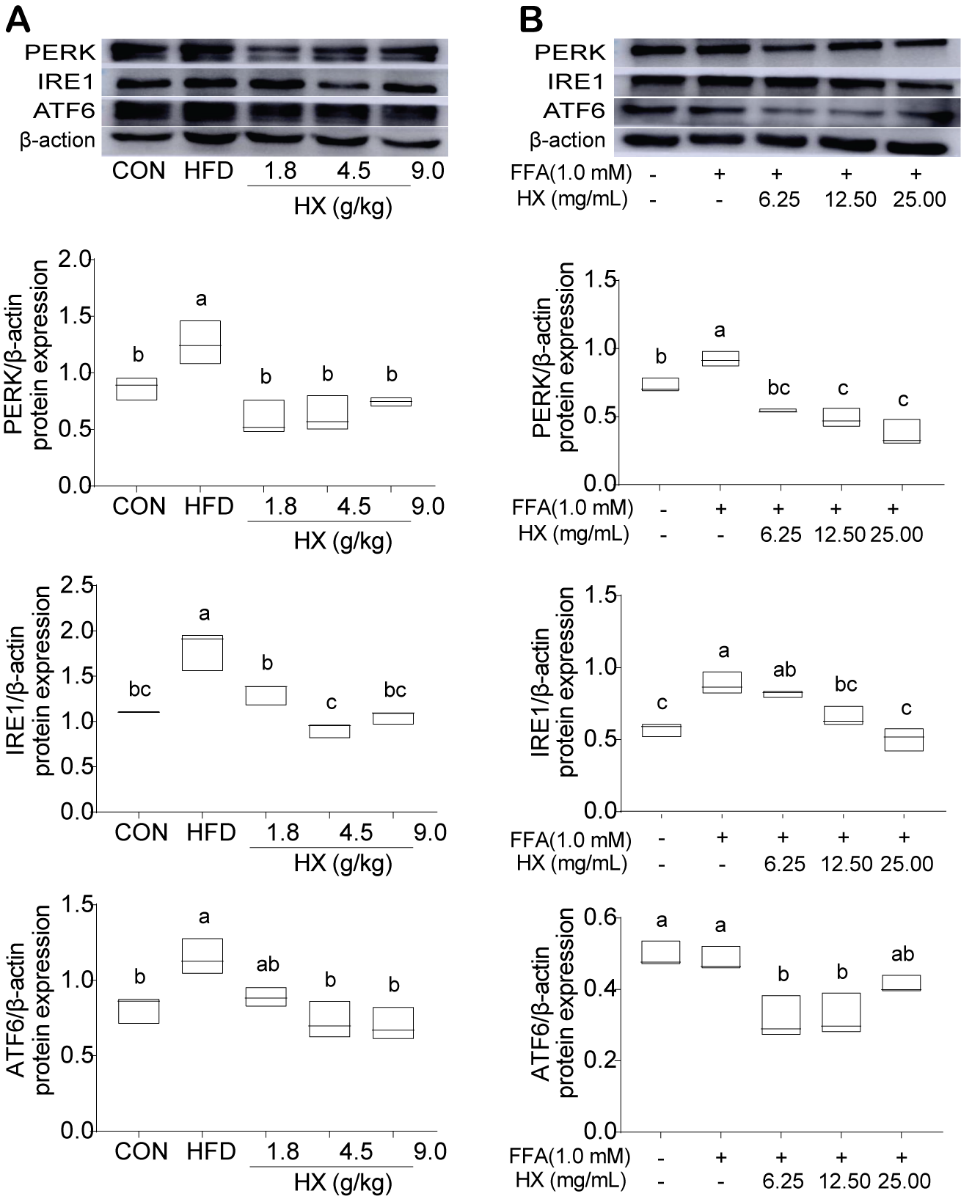


**Supplementary Figure S3.** Abundance of endoplasmic reticulum stress (ERS)-related proteins in liver tissue (panel A) of mice after 8 weeks of HFD feeding and AML12 cells incubated without or with FFA and different concentrations of HX (6.25, 12.50, and 25.00 mg/mL) (panel B). Data were analyzed by a one-way ANOVA and multiple comparisons was employed, and results are expressed as the mean ± SEM (n=6 per group). Statistical analyses were performed using Student’s *t* test. Different lowercase letters a, b and c denote significant differences between any two groups (*P* < 0.05), but with the same lowercase letters, like bc and c, ab and a or b, ab and bc means no differences between the two groups (*P* > 0.05). PERK, protein kinase PKR-like endoplasmic reticulum kinase; IRE1, inositol-requiring enzyme-1; ATF6, transcription factor 6; CON, control; HFD, high-fat diet; FFA, free fatty acid; HX, Huo Xiang.

**Supplementary Table**

**Supplementary Table S1 Diet Formulae^1^**

| Diet composition | CON | HFD |
| --- | --- | --- |
| Ingredients, *g/kg* |  |  |
| Casein | 140.0 | 172.8 |
| Corn starch | 495.7 | 34.0 |
| Maltodextrin 10 | 125.0 | 123.5 |
| Sucrose | 100.0 | 308.6 |
| Cellulose | 50.0 | 61.7 |
| L-Cystine | 1.8 | 2.2 |
| Mineral mix^2^ | 35.0 | 43.2 |
| Vitamin mix^3^ | 10.0 | 12.3 |
| Choline bitartrate | 2.5 | 3.1 |
| Corn oil | 40.0 | 49.4 |
| Lard | -- | 188.9 |
| Energy density, *kcal/g* | 3.85 | 4.76 |
| Energy percentage, % |  |  |
| Protein | 14.7 | 14.7 |
| Carbohydrate | 75.9 | 40.3 |
| Fat | 9.4 | 45.0 |

^1^CON, control diet; HFD, high-fat diet.

^2^Mineral mix for AIN-93M diet.

^3^Vitamin mix for AIN-93 diet.

**Supplementary Table S2. Chemical components identiﬁed in AR by high-performance liquid chromatography-electrospray ionization/mass spectrometry (ESI +)**

| Name | Rt [min] | Molecular Weight | CAS | Content (ng/μL) | |
| --- | --- | --- | --- | --- | --- |
| L-Phenylalanine | 2.983 | 165.0785 | 63-91-2 | | 20.55 |
| L-Arginine | 1.201 | 174.111 | 74-79-3 | | 6.54 |
| L-Tyrosine | 1.397 | 181.0732 | 60-18-4 | | 0.26 |
| L-Glutamate | 1.313 | 147.0525 | 56-86-0 | | 0.18 |
| L-Isoleucine | 2.072 | 131.0941 | 61-90-5 | | 29.93 |
| L-Lysine | 1.16 | 146.1049 | 56-87-1 | | 0.35 |
| L-Proline | 1.341 | 115.0628 | 147-85-3 | | 2.27 |
| Pyroglutamic acid | 1.98 | 129.042 | 98-79-3 | | 8.26 |
| ferulic acid | 4.69 | 194.0573 | 1135-24-6 | | 14.81 |
| Sinapic acid | 4.66 | 224.0675 | 530-59-6 | | 0.83 |
| Styrene | 6.038 | 104.0621 | 100-42-5 | | 0.10 |
| Chorismic acid | 3.906 | 226.0474 | 617-12-9 | | 0.03 |
| m-Coumaric acid | 4.64 | 164.0467 | 588-30-7 | | 2.13 |
| 1,2,3-Trihydroxybenzene | 3.232 | 126.0311 | 533-73-3 | | 1.47 |
| Caffeic Acid | 4.48 | 180.0415 | 4607-41-4 | | 3.61 |
| Thymol | 4.451 | 150.1038 | 89-83-8 | | 0.71 |
| Adenosine | 1.965 | 267.0958 | 58-61-7 | | 3.88 |
| Adenine | 1.957 | 135.0546 | 73-24-5 | | 0.01 |
| Guanosine | 1.96 | 283.0908 | 118-00-3 | | 1.44 |
| Guanine | 1.441 | 151.0487 | 73-40-5 | | 1.96 |
| cAMP | 1.473 | 329.0502 | 60-92-4 | | 6.56 |
| Quercetin 3-galactoside | 4.491 | 464.0946 | 482-36-0 | | 0.06 |
| Arcapillin | 5.282 | 360.0832 | NA | | 0.21 |
| Glyceollin | 5.928 | 338.1144 | NA | | 0.01 |
| Isorhamnetin | 4.954 | 316.0571 | 480-19-3 | | 0.20 |
| Malvidin | 5.267 | 330.0727 | 643-84-5 | | 0.04 |
| Naringenin | 5.177 | 272.0675 | 480-41-1 | | 0.06 |
| Quercetin | 4.988 | 302.0415 | 117-39-5 | | 0.09 |
| Quercetin 3-(3-p-coumaroylglucoside) | 4.653 | 610.1301 | 76211-70-6 | | 0.03 |
| Rhamnetin | 5.528 | 316.0572 | 480-19-3 | | 2.23 |
| Taxifolin | 4.405 | 304.0572 | 480-18-2 | | 0.05 |
| Cyanidin 3-O-rutinoside | 4.334 | 594.1558 | 28338-59-2 | | 9.62 |
| Diosmetin | 5.179 | 300.0621 | 520-34-3 | | 0.95 |
| Eriodictyol | 4.51 | 288.0621 | 552-58-9 | | 0.09 |
| Genistein | 4.478 | 270.0516 | 446-72-0 | | 3.98 |
| Genistin | 4.476 | 432.1037 | 529-59-9 | | 1.52 |
| Luteolin | 4.806 | 286.0465 | 491-70-3 | | 2.03 |
| Pelargonidin 3-O-(6-O-malonyl-β-D-glucoside) | 4.525 | 518.1035 | 165070-68-8 | | 0.04 |
| Pelargonidin 3-O-rutinoside | 4.389 | 578.1612 | NA | | 0.05 |
| Petunidin 3-O-glucoside | 4.537 | 478.1092 | 6988-81-4 | | 0.05 |
| Quercitrin | 4.503 | 448.0987 | 522-12-3 | | 0.53 |
| Sakuranin | 4.532 | 448.1351 | NA | | 0.06 |
| Scutellarein 5-glucuronide | 4.501 | 462.0778 | NA | | 5.96 |
| Naringin | 4.482 | 580.1763 | 10236-47-2 | | 0.02 |
| Gallocatechin | 1.388 | 306.0707 | NA | | 0.10 |
| Peonidin 3-rhamnoside 5-glucoside | 13.76 | 609.1748 | 53859-11-3 | | 0.08 |
| Hesperetin | 4.538 | 302.0778 | 520-33-2 | | 0.13 |
| 2-Hexyl-3-phenyl-2-propenal | 5.773 | 216.1506 | 101-86-0 | | 20.99 |
| DL-pipecolic acid | 1.925 | 129.0785 | 535-75-1 | | 1.21 |
| Hydroquinidine | 4.963 | 326.1984 | 1435-55-8 | | 0.02 |
| Hypoxanthine | 1.963 | 136.0379 | 68-94-0 | | 1.48 |
| Trigonelline | 1.584 | 137.0471 | 535-83-1 | | 11.84 |
| Xanthosine | 4.474 | 284.0787 | 146-80-5 | | 0.11 |
| Caffeine | 4.413 | 194.0837 | 58-08-2 | | 0.07 |
| D-Mannitol | 1.231 | 182.0785 | 69-65-8 | | 0.18 |
| a-L-Rhamnose | 1.239 | 164.0679 | 6014-42-2 | | 0.08 |
| Gibberellin A53 | 5.402 | 348.1923 | NA | | 0.12 |
| Glutinosone | 5.699 | 220.1455 | 55051-94-0 | | 13.78 |
| Plaunol B | 4.789 | 356.1247 | 69749-00-4 | | 1.01 |
| Quillaic acid | 6.58 | 486.3329 | 631-01-6 | | 0.03 |
| Genipin | 4.406 | 226.083 | 6902-77-8 | | 0.34 |
| Medicagenic acid | 6.215 | 502.327 | 599-07-5 | | 0.08 |
| p-Cymene | 4.894 | 134.1089 | NA | | 0.18 |
| Pantothenic Acid | 3.524 | 219.1103 | 137-08-6 | | 10.65 |
| Pyridoxine | 2.326 | 169.0736 | 65-23-6 | | 1.34 |
| Pyridoxal | 3.258 | 167.0579 | 66-72-8 | | 0.08 |
| Niacin | 5.633 | 123.0314 | 59-67-6 | | 0.04 |
| Niacinamide | 1.985 | 122.0473 | 98-92-0 | | 1.87 |
| Palmitic amide | 9.57 | 255.2558 | 629-54-9 | | 1.77 |
| 13Z-Docosenamide | 13.06 | 337.3334 | 112-84-5 | | 12.71 |
| Oleamide | 9.873 | 281.2709 | 301-02-0 | | 4.09 |
| Stearamide | 12.982 | 283.2865 | 124-26-5 | | 1.02 |
| Coumarin | 5.111 | 146.0362 | 91-64-5 | | 0.13 |
| 3 Hydroxycoumarin | 3.902 | 162.0309 | 939-19-5 | | 1.08 |
| Scopoletin | 4.766 | 192.0414 | NA | | 0.25 |
| Benzoic acid | 4.7 | 122.0362 | 65-85-0 | | 0.59 |
| α-ketoisovaleric acid | 1.86 | 116.0469 | 759-05-7 | | 0.70 |
| Succinic acid | 1.957 | 118.0273 | 110-15-6 | | 5.02 |
| nandrolone | 5.468 | 274.1923 | 434-22-0 | | 1.10 |
| α-Linolenic Acid | 7.357 | 278.224 | 463-40-1 | | 3.14 |
| Butyric acid | 1.866 | 88.0521 | 107-92-6 | | 2.91 |
| LysoPC(16:0) | 7.257 | 495.3313 | NA | | 0.95 |
| MG(0:0/18:3/0:0) | 6.214 | 352.2602 | NA | | 0.09 |
| Indoleacrylic acid | 4.278 | 187.0625 | 1204-06-4 | | 2.79 |
| Methyl cinnamate | 3.805 | 162.0675 | 103-26-4 | | 0.26 |
| 5-Hydroxy-L-tryptophan | 2.276 | 220.0845 | 4350-09-8 | | 0.26 |
| Indoleacetaldehyde | 2.371 | 159.0681 | NA | | 0.05 |
| Acetylcholine | 2.005 | 145.1099 | 51-84-3 | | 3.80 |
| Cinnamic acid | 3.612 | 148.0521 | 621-82-9 | | 0.22 |
| Gingerol | 5.765 | 294.182 | 58253-27-3 | | 1.39 |
| Hippuric acid | 4.356 | 179.0576 | 495-69-2 | | 0.10 |
| Jasmolone | 5.898 | 180.1144 | 54383-66-3 | | 1.12 |
| (-)-Jasmonic acid | 5.713 | 210.1247 | 6894-38-8 | | 10.95 |
| Indole | 4.301 | 117.0573 | 120-72-9 | | 20.21 |
| Methyl jasmonate | 4.519 | 224.1403 | 39924-52-2 | | 7.52 |
| Phenylacetic acid | 4.746 | 136.0518 | 103-82-2 | | 0.81 |
| acetophenone | 4.403 | 120.0568 | 98-86-2 | | 0.44 |
| Choline | 9.289 | 103.0991 | 62-49-7 | | 0.03 |
| Tropic acid | 4.458 | 166.065 | 552-63-6 | | 0.60 |

**Supplementary Table S3. Chemical components identiﬁed in AR by high-performance liquid chromatography-electrospray ionization/mass spectrometry (ESI -)**

| Name | Rt [min] | Molecular Weight | CAS | Content (ng/μL) |
| --- | --- | --- | --- | --- |
| L-Isoleucine | 2.06 | 131.09469 | 61-90-5 | 34.52 |
| L-Phenylalanine | 2.933 | 165.07893 | 63-91-2 | 211.45 |
| Pyroglutamic acid | 1.991 | 129.04272 | 98-79-3 | 401.32 |
| L-Cystine | 4.179 | 240.02653 | 56-89-3 | 5.94 |
| Chlorogenic Acid | 4.127 | 354.09478 | 327-97-9 | 1.41 |
| ferulic acid | 4.705 | 194.0574 | 1135-24-6 | 26.94 |
| Sinapic acid | 4.68 | 224.06787 | 530-59-6 | 11.20 |
| 1,2,3-Trihydroxybenzene | 3.154 | 126.03172 | 533-73-3 | 8.90 |
| Caffeic Acid | 3.013 | 180.04208 | 4607-41-4 | 3.41 |
| Gallic acid | 3.708 | 170.02138 | 149-91-7 | 6.94 |
| Gentisic acid | 3.623 | 154.0266 | 490-79-9 | 88.81 |
| Shikimic acid | 1.836 | 174.05273 | 138-59-0 | 2.73 |
| Homogentisic acid | 3.694 | 168.04204 | 451-13-8 | 209.40 |
| m-Coumaric acid | 4.65 | 164.04712 | 588-30-7 | 148.77 |
| Syringic acid | 2.887 | 198.05249 | 530-57-4 | 7.29 |
| Salicylic acid | 4.496 | 138.03141 | 69-72-7 | 132.00 |
| Uridine | 2.02 | 244.06907 | 58-96-8 | 22.44 |
| Inosine | 1.276 | 268.07889 | 58-63-9 | 33.93 |
| IMP | 4.452 | 348.04661 | 131-99-7 | 4.17 |
| cAMP | 1.971 | 329.05183 | 60-92-4 | 9.29 |
| Diosmetin | 5.179 | 300.06245 | 520-34-3 | 7.96 |
| Genistein | 4.566 | 270.05208 | 446-72-0 | 37.27 |
| Malvidin | 5.272 | 330.07307 | 643-84-5 | 2.78 |
| Naringenin | 5.185 | 272.06776 | 480-41-1 | 4.14 |
| Quercetin | 5.038 | 302.04179 | 117-39-5 | 3.87 |
| Cyanidin 3-O-rutinoside | 4.326 | 594.15626 | 28338-59-2 | 192.70 |
| Isorhamnetin | 4.948 | 316.05741 | 480-19-3 | 1.67 |
| Luteolin | 4.861 | 286.04682 | 491-70-3 | 45.32 |
| Pelargonidin 3-O-rutinoside | 4.944 | 578.16133 | NA | 0.98 |
| Petunidin 3-O-glucoside | 4.585 | 478.10942 | 6988-81-4 | 3.95 |
| Quercitrin | 4.555 | 448.09913 | 522-12-3 | 7.41 |
| Dihydromyricetin | 4.479 | 320.05192 | 27200-12-0 | 1.21 |
| Eriodictyol | 4.523 | 288.06209 | 552-58-9 | 0.94 |
| Naringin | 4.499 | 580.17667 | 10236-47-2 | 0.95 |
| Quercetin 3-(3-p-coumaroylglucoside) | 4.67 | 610.12941 | 76211-70-6 | 1.07 |
| Quercetin 3-galactoside | 4.519 | 464.09335 | 482-36-0 | 1.39 |
| Scutellarein 5-glucuronide | 4.502 | 462.07786 | NA | 63.58 |
| Taxifolin | 4.43 | 304.05702 | 480-18-2 | 1.27 |
| Rutin | 4.428 | 610.14931 | 153-18-4 | 1.61 |
| Hesperetin | 4.523 | 302.07789 | 520-33-2 | 1.59 |
| Purine | 1.299 | 120.04223 | 120-73-0 | 349.43 |
| 2-Furoic acid | 1.439 | 112.01615 | 88-14-2 | 241.17 |
| Caffeine | 4.492 | 194.08423 | 58-08-2 | 1.85 |
| D-Glucarate | 1.543 | 210.03737 | 87-73-0 | 63.10 |
| D-Glucuronic acid | 1.264 | 194.04247 | 6556-12-3 | 45.47 |
| Glutaric acid | 1.311 | 132.04226 | 110-94-1 | 235.59 |
| L-Xylulose | 1.458 | 150.05294 | 527-50-4 | 44.41 |
| D-Mannitol | 1.265 | 182.07878 | 69-65-8 | 140.84 |
| Gluconic acid | 1.299 | 196.058 | 526-95-4 | 1538.85 |
| α-D-Glucose | 1.307 | 180.06317 | 492-62-6 | 990.57 |
| α,α-Trehalose | 1.738 | 342.1154 | 57-50-1 | 65.82 |
| Raffinose | 4.067 | 504.16731 | 512-69-6 | 1.87 |
| Genipin | 4.414 | 226.08368 | 6902-77-8 | 18.67 |
| Gibberellin A12 | 8.093 | 332.19787 | NA | 1.67 |
| Medicagenic acid | 6.193 | 502.32825 | 599-07-5 | 11.66 |
| Quillaic acid | 6.564 | 486.33328 | 631-01-6 | 2.14 |
| Rishitin | 7.443 | 222.16141 | 18178-54-6 | 38.69 |
| Gibberellin A17 | 4.924 | 378.1664 | 18411-79-5 | 3.45 |
| Gibberellin A36 | 5.465 | 362.17181 | NA | 1.11 |
| Ganoderic acid H | 17.348 | 572.2945 | 98665-19-1 | 20.01 |
| Geranyl diphosphate | 4.391 | 314.06284 | 763-10-0 | 48.34 |
| Pantothenic Acid | 3.485 | 219.1103 | 137-08-6 | 383.90 |
| Riboflavin | 4.246 | 376.1359 | 83-88-5 | 22.96 |
| Sulfuric acid | 1.575 | 97.96744 | 7664-93-9 | 1453.79 |
| Phosphoric acid | 1.471 | 97.97696 | 7664-38-2 | 170.76 |
| Benzoic acid | 4.717 | 122.03673 | 65-85-0 | 79.39 |
| Citric acid | 1.446 | 192.02674 | 77-92-9 | 2694.59 |
| Lactic acid | 2.959 | 90.0318 | 50-21-5 | 15.36 |
| Pyruvate | 1.45 | 88.01615 | 127-17-3 | 92.66 |
| Hexadecanedioic acid | 5.656 | 286.21382 | NA | 15.38 |
| Quinic acid | 4.373 | 192.06302 | 77-95-2 | 14.95 |
| Aconitic acid | 2 | 174.0164 | 499-12-7 | 178.17 |
| Itaconic acid | 2.512 | 130.02669 | 97-65-4 | 283.47 |
| Maleic acid | 1.996 | 116.01102 | 110-16-7 | 272.96 |
| Malic acid | 1.879 | 134.02155 | 6915-15-7 | 1291.47 |
| Oxoglutaric acid | 1.487 | 146.02162 | 328-50-7 | 20.79 |
| Succinic acid | 2.072 | 118.02664 | 110-15-6 | 1453.94 |
| Glyceric acid | 1.354 | 106.02678 | 473-81-4 | 85.25 |
| nandrolone | 5.46 | 274.19264 | 434-22-0 | 1.28 |
| α-Linolenic Acid | 7.321 | 278.22397 | 463-40-1 | 164.66 |
| LysoPC(15:0) | 7.22 | 481.31539 | NA | 39.74 |
| Traumatic Acid | 5.273 | 228.13561 | 6402-36-4 | 49.42 |
| acetophenone | 4.646 | 120.05742 | 98-86-2 | 94.66 |
| Citramalic acid | 1.499 | 148.03727 | 2306-22-1 | 110.90 |
| Mevalonic acid | 3.028 | 148.07363 | 150-97-0 | 74.37 |
| Phenylacetic acid | 4.741 | 136.05243 | 103-82-2 | 24.37 |
| (-)-Jasmonic acid | 5.711 | 210.12533 | 6894-38-8 | 378.66 |
| Malonic acid | 1.474 | 104.0111 | 141-82-2 | 566.06 |
| Xanthoxin | 6.275 | 250.15644 | 8066-07-07 | 259.70 |
| Gentisin | 4.621 | 258.05214 | 437-50-3 | 5.05 |
| Tropic acid | 4.432 | 166.06257 | 552-63-6 | 5.88 |
| Xanthoxic acid | 9.992 | 266.15443 | NA | 7.21 |

**Supplementary Table S4 Potential active components of patchouli ranked by degree (OB ≥ 30% or DL ≥ 0.18)**

| ID | MOL ID | Potential active compounds | OB (%) | DL |
| --- | --- | --- | --- | --- |
| HX1 | MOL005911 | 5-Hydroxy-7,4'-dimethoxyflavanon | 51.54 | 0.27 |
| HX2 | MOL005921 | quercetin 7-O-β-D-glucoside | 49.57 | 0.27 |
| HX3 | MOL000098 | quercetin | 46.43 | 0.28 |
| HX4 | MOL002879 | Diop | 43.59 | 0.39 |
| HX5 | MOL005922 | Acanthoside B | 43.35 | 0.77 |
| HX6 | MOL005918 | phenanthrone | 38.70 | 0.33 |
| HX7 | MOL005916 | irisolidone | 37.78 | 0.30 |
| HX8 | MOL005573 | Genkwanin | 37.13 | 0.24 |
| HX9 | MOL005923 | 3,23-dihydroxy-12-oleanen-28-oic acid | 30.86 | 0.86 |

**Supplementary Table S5 Top 20 clusters with their representative enriched terms (one per cluster)**

| GO | Category | Description | Count | % | Log10(P) | Log10(q) |
| --- | --- | --- | --- | --- | --- | --- |
| ko05418 | KEGG Pathway | Fluid shear stress and atherosclerosis | 25 | 30.49 | -37.63 | -34.99 |
| ko04933 | KEGG Pathway | AGE-RAGE signaling pathway in diabetic complications | 23 | 28.05 | -37.59 | -34.99 |
| hsa05200 | KEGG Pathway | Pathways in cancer | 31 | 37.8 | -35.67 | -33.47 |
| hsa05162 | KEGG Pathway | Measles | 17 | 20.73 | -21.58 | -20 |
| hsa04066 | KEGG Pathway | HIF-1 signaling pathway | 15 | 18.29 | -20.11 | -18.68 |
| ko05146 | KEGG Pathway | Amoebiasis | 14 | 17.07 | -19.71 | -18.3 |
| hsa04932 | KEGG Pathway | Non-alcoholic fatty liver disease (NAFLD) | 16 | 19.51 | -19.39 | -18.04 |
| hsa04151 | KEGG Pathway | PI3K-Akt signaling pathway | 19 | 23.17 | -17.76 | -16.51 |
| hsa04919 | KEGG Pathway | thyroid hormone signaling pathway | 12 | 14.63 | -14.75 | -13.65 |
| hsa04931 | KEGG Pathway | insulin resistance | 11 | 13.41 | -13.54 | -12.49 |
| hsa05020 | KEGG Pathway | Prion diseases | 8 | 9.76 | -13.1 | -12.09 |
| hsa01524 | KEGG Pathway | Platinum drug resistance | 9 | 10.98 | -12.1 | -11.14 |
| hsa04611 | KEGG Pathway | Platelet activation | 10 | 12.2 | -11.3 | -10.36 |
| hsa04211 | KEGG Pathway | Longevity regulating pathway | 9 | 10.98 | -10.49 | -9.61 |
| hsa05202 | KEGG Pathway | Transcriptional misregulation in cancer | 10 | 12.2 | -9.36 | -8.55 |
| hsa04630 | KEGG Pathway | Jak-STAT signaling pathway | 8 | 9.76 | -7.73 | -7.03 |
| hsa04670 | KEGG Pathway | Leukocyte transendothelial migration | 7 | 8.54 | -7.36 | -6.68 |
| hsa05211 | KEGG Pathway | Renal cell carcinoma | 5 | 6.1 | -5.88 | -5.26 |
| hsa04930 | KEGG Pathway | Type II diabetes mellitus | 4 | 4.88 | -5 | -4.45 |
| hsa03320 | KEGG Pathway | PPAR signaling pathway | 4 | 4.88 | -4.23 | -3.73 |
